# Delayed Postoperative Malignant Hyperthermia After Total Intravenous Anesthesia: A Case Report

**DOI:** 10.7759/cureus.115867

**Published:** 2026-09-06

**Authors:** Jeongeun Lee, Hyungtae Kim, Won Uk Koh, Jaedo Lee, Seeun Jung

**Affiliations:** 1 Department of Anesthesiology and Pain Medicine, Veterans Health Service Medical Center, Seoul, KOR; 2 Department of Anesthesiology, Asan Medical Center, Seoul, KOR

**Keywords:** dantrolene, malignant hyperthermia, muscle rigidity, postoperative complication, ryanodine receptor, total intravenous anesthesia

## Abstract

Malignant hyperthermia (MH) is a rare pharmacogenetic disorder typically triggered by volatile anesthetics or succinylcholine, but its occurrence after total intravenous anesthesia (TIVA) remains exceptional and underreported. Propofol-based TIVA is recommended for MH-susceptible patients to avoid known triggers, yet absolute prevention cannot be guaranteed.

A 24-year-old male with prior suspected MH underwent spine surgery under propofol-remifentanil TIVA after strict preventive preparation. Despite stable intraoperative parameters, he developed fever, tachycardia, rigidity, and myoglobinuria 14 hours postoperatively, with markedly elevated creatine kinase and myoglobin levels. Dantrolene administration resulted in rapid clinical improvement. The Malignant Hyperthermia Clinical Grading Scale score of 48 indicated “very likely MH.”

This case underscores the risk of MH even with TIVA in susceptible patients, emphasizing the need for vigilant monitoring, availability of dantrolene, and preparedness for delayed onset regardless of the anesthetic technique used.

## Introduction

Malignant hyperthermia (MH) is a rare but potentially fatal pharmacogenetic muscle disorder triggered by volatile anesthetics or depolarizing muscle relaxants in genetically susceptible individuals due to dysregulated intracellular calcium homeostasis [1]. Clinical manifestations, including severe muscle rigidity, hyperthermia, tachycardia, and acidosis, require rapid diagnosis and dantrolene administration [2]. While volatile anesthetics are well-known triggers, propofol-based total intravenous anesthesia (TIVA) is recommended for MH-susceptible patients because it avoids known anesthetic triggers [3]. However, rare MH-like episodes without exposure to known anesthetic triggers, including delayed postoperative presentations, have been reported. MH susceptibility can be evaluated by genetic testing or muscle contracture testing. This case describes a patient with a previous history of suspected MH who developed a delayed postoperative hypermetabolic episode after trigger-free TIVA.

## Case presentation

A 24-year-old male with congenital scoliosis (height = 152 cm, weight = 41 kg) was scheduled for spine surgery (deformity correction, posterior spinal fusion). He had previously undergone general anesthesia for left knee dislocation surgery in June 2018 at another hospital, during which his body temperature rose sharply, leading to early termination of that procedure due to suspected MH. Since genetic testing was not performed, a genetic diagnosis could not be confirmed, but he was nevertheless considered at high risk for MH.

In preparation for anesthesia in this patient suspected of having MH, preventive measures were implemented according to the institutional protocol of Asan Medical Center. Inhalation anesthetic agents and succinylcholine were strictly avoided, and a TIVA technique using target effect-site concentrations of propofol 2-3 µg/mL and remifentanil 5-10 ng/mL was administered instead to prevent exposure to MH-triggering agents. The case was scheduled as the first procedure of the day to minimize exposure risk, and the intensive care unit was notified and prepared to receive the patient postoperatively if necessary. However, since complete prevention cannot be guaranteed, the patient was informed about the potential risk of MH during surgery and the possibility of fatal outcomes. The availability of dantrolene, the specific treatment for MH, was confirmed by phone, with 18 vials in stock and additional supply obtainable if needed.

During the operation, there were no remarkable events. The patient's vital signs remained stable throughout the procedure, with a temperature ranging from 35.4 to 36.8℃, heart rate between 52 and 72 beats per minute, blood pressure consistently around 98/52 to 99/65 mmHg, end-tidal CO₂ levels from 31 to 38 mmHg, oxygen saturation (SpO₂) maintained at 100%, and respiratory rate between 10 and 12 breaths per minute.

Fourteen hours postoperatively, the patient developed fever, tachycardia, muscle rigidity, and hematuria. Vital signs showed blood pressure of 105/70 mmHg, heart rate of 115 beats/min, respiratory rate of 32 breaths/min, and temperature of 38.4℃. Laboratory tests revealed markedly elevated creatine kinase (3339 IU/L), blood myoglobin (925 ng/mL), urinary myoglobin (296 ng/mL), and lactate (1.5 mmol/L), and mild coagulopathy (prothrombin time-international normalized ratio (PT-INR) = 1.48). Arterial blood gas analysis showed a pH of 7.413, partial pressure of carbon dioxide (pCO₂) of 46.2 mmHg, bicarbonate (HCO₃⁻) of 29.4 mmol/L, and partial pressure of oxygen (pO₂) of 72.3 mmHg. The patient showed cola-colored urine (Figure 1).

**Figure 1:**
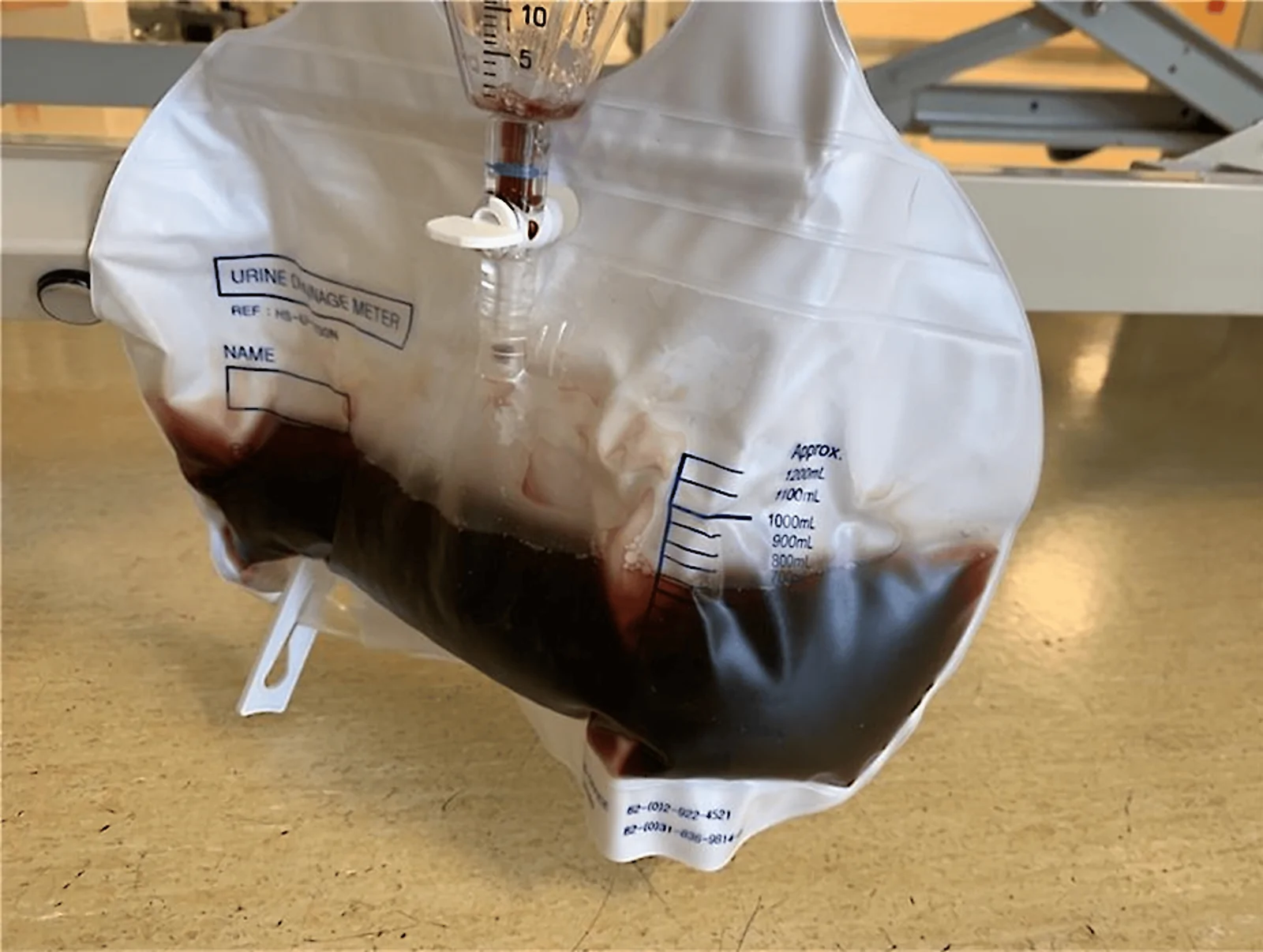
Cola-colored urine indicating hematuria was observed 14 hours postoperatively in the malignant hyperthermia patient.

Intravenous dantrolene 40 mg was initially administered, followed by an additional 40 mg, resulting in a cumulative dose of 80 mg (approximately 2.0 mg/kg). Following treatment, laboratory parameters improved progressively, with creatine kinase (CK) decreasing to 1017 IU/L over three days, blood myoglobin to 18 ng/mL, urinary myoglobin to 77 ng/mL, and lactate dehydrogenase (LD) to 331 IU/L, indicating recovery. Vital signs stabilized to 113/64 mmHg, 105 beats/min, 17 breaths/min, and 37.3℃.

The patient achieved a total Malignant Hyperthermia Clinical Grading Scale (MHCGS) score of 48 points, which corresponds to "very likely malignant hyperthermia," according to the established scale (Table 1) [4].

**Table 1:** Malignant Hyperthermia Clinical Grading Scale criteria and patient-specific findings. Clinical indicators of the Malignant Hyperthermia Clinical Grading Scale (MHCGS), corresponding findings in this patient, and points assigned are presented. The total MHCGS score was 48, corresponding to rank 5 (“very likely” malignant hyperthermia). For each clinical process, multiple indicators were not scored cumulatively beyond the maximum score assigned to that process. PETCO2: partial pressure of end-tidal carbon dioxide; PaCO2: partial pressure of carbon dioxide. Adapted from Larach et al. [4].

Process	Indicators	Points	Findings in this patient	Points assigned
Process I: Rigidity	Generalized muscular rigidity (in the absence of shivering due to hypothermia, or during/immediately following emergence from inhalational general anesthesia)	15	Generalized muscle rigidity 14 hours postoperatively	15
Process I: Rigidity	Masseter spasm shortly following succinylcholine administration	15	.	
Process II: Muscle Breakdown	Elevated creatine kinase >20,000 IU/L after anesthetic that included succinylcholine	15	.	
Process II: Muscle Breakdown	Elevated creatine kinase >10,000 IU/L after anesthetic without succinylcholine	15	.	
Process II: Muscle Breakdown	Cola-colored urine in perioperative period	10	Cola-colored urine 14 hours postoperatively	10
Process II: Muscle Breakdown	Myoglobin in urine >60 ng/mL	5	Urinary myoglobin = 296 ng/mL	5
Process II: Muscle Breakdown	Myoglobin in serum >170 ng/mL	5	Serum myoglobin = 925 ng/mL	5
Process II: Muscle Breakdown	Blood/plasma/serum K+ >6 mEq/L (in the absence of renal failure)	3	.	
Process III: Respiratory Acidosis	PETCO2 >55 mmHg with appropriately controlled ventilation	15	.	
Process III: Respiratory Acidosis	Arterial PaCO2 >60 mmHg with appropriately controlled ventilation	15	PaCO₂ of 46.2 mmHg	
Process III: Respiratory Acidosis	PETCO2 >60 mmHg with spontaneous ventilation	15	.	
Process III: Respiratory Acidosis	Arterial PaCO2 >65 mmHg with spontaneous ventilation	15	.	
Process III: Respiratory Acidosis	Inappropriate hypercarbia (anesthesiologist's judgment)	15	.	
Process III: Respiratory Acidosis	Inappropriate tachypnea	10	Respiratory rate of 32 breaths/min	10
Process IV: Temperature Increase	Inappropriately rapid increase in temperature (anesthesiologist's judgment)	15	.	
Process IV: Temperature Increase	Inappropriately increased temperature >38.8°C (101.8°F) in perioperative period	10	Temperature of 38.4°C at symptom onset	
Process V: Cardiac Involvement	Inappropriate sinus tachycardia	3	Heart rate of 115 beats/min	3
Process V: Cardiac Involvement	Ventricular tachycardia or ventricular fibrillation	3	.	
Other indicators	Arterial base excess more negative than -8 mEq/L	10	.	
Other indicators	Arterial pH <7.25	10	.	
Other indicators	Rapid reversal of malignant hyperthermia signs of metabolic and/or respiratory acidosis with IV dantrolene	5	Clinical improvement following IV dantrolene administration	5

## Discussion

MH is a pharmacogenetic disorder characterized by uncontrolled intracellular calcium release in skeletal muscle due to mutations in the ryanodine receptor (RYR1) or other related genes [5,6]. This leads to a hypermetabolic state triggered typically by volatile anesthetics or depolarizing muscle relaxants. The excessive calcium influx causes sustained muscle contraction, hypercapnia, acidosis, hyperthermia, and rhabdomyolysis.

However, MH is classically associated with volatile agents; its occurrence after TIVA using non-triggering agents like propofol and remifentanil is almost unreported [7]. Although multiple case reports have described TIVA as safe in patients susceptible to MH, the present case highlights that TIVA may not completely prevent MH [8,9]. This finding offers important clinical implications, suggesting the possibility of MH occurrence even with non-triggering anesthetic techniques like TIVA, and indicating that factors such as genetic susceptibility or unidentified triggers may also contribute.

The MHCGS provides an objective method to estimate the likelihood of MH based on clinical signs and lab findings, aiding diagnosis without specialized tests [10]. However, it may underestimate MH in incomplete or atypical cases and should be used alongside clinical judgment [4]. This grading scale encompasses six main symptom groups. In the present case, the observed clinical features included generalized muscular rigidity, cola-colored urine in the perioperative period, inappropriate hypercarbia as assessed by the anesthesiologist, and inappropriate sinus tachycardia. The elevated creatine kinase and myoglobin confirmed significant muscle breakdown, consistent with MH, and that myoglobinuria can risk acute kidney injury. With a total score of 48 points on the MHCGS, the patient is classified as "very likely malignant hyperthermia," according to the validated scoring system. Diagnosis of MH was made by combining the MHCGS score with clinical judgment.

Postoperative rhabdomyolysis related to prolonged spine surgery was considered an important alternative diagnosis. Other causes of hypermetabolism, including sepsis, thyroid storm, pheochromocytoma, neuroleptic malignant syndrome, and serotonin syndrome, were also considered. However, the combination of muscle rigidity, tachycardia, myoglobinuria, and the previous history of suspected MH made MH the most likely clinical diagnosis.

Non-anesthetic factors such as pain, physical or emotional stress, and shivering have also been suggested as possible triggers of MH-like episodes in susceptible patients [11]. Although a specific trigger could not be identified in this case, these factors may be relevant to the delayed postoperative presentation.

Prolonged surgery in the prone position was considered as an alternative cause of postoperative rhabdomyolysis. However, rhabdomyolysis alone did not account for the overall clinical presentation, particularly the concurrent postoperative hyperthermia and tachycardia, which raised suspicion for MH. Therefore, MH was considered based on the overall clinical constellation rather than the biochemical evidence of muscle injury alone.

The clinical significance of this case lies in the recognition that MH should be considered even during TIVA protocols, especially in patients with prior suspicious history or family history suggestive of MH susceptibility. Despite preventive measures, including preparation of dantrolene and postoperative ICU monitoring, a critical MH crisis occurred, highlighting the importance of early recognition and prompt management. Thus, anesthesia providers must remain vigilant for signs of MH and be prepared to initiate immediate intervention by securing dantrolene availability, even when using TIVA.

Moreover, compared with previous reports, this case is also noteworthy due to the delayed onset of MH symptoms approximately 14 hours after surgery, which is unusual but has been documented in the literature [12,13]. It should be noted that MH can occur even when not immediately after surgery. Preventive measures include thorough preoperative assessment for MH risk factors, avoidance of known trigger agents, readiness to administer dantrolene promptly, and continuous education of anesthesia personnel. Further research is warranted to elucidate the pathophysiology of MH in non-triggering anesthetic settings and to improve diagnostic protocols for atypical presentations.

The main limitations of this case are the absence of genetic testing to confirm MH susceptibility and the lack of detailed information regarding previous anesthetic exposure, including the degree of perioperative hyperthermia and associated symptoms. Further research involving genetic analysis and detailed retrospective evaluation of previous anesthetic exposures would help strengthen the understanding of MH presentations during TIVA and contribute to ensuring the patient’s safety in future surgical procedures.

## Conclusions

This case highlights that MH may occur despite the use of TIVA with non-triggering agents in patients who are susceptible to MH. The delayed postoperative presentation in this patient emphasizes that the absence of intraoperative abnormalities does not completely exclude the diagnosis. Early recognition of clinical signs, including muscle rigidity, tachycardia, hyperthermia, and myoglobinuria, allowed prompt administration of dantrolene and favorable recovery. Anesthesiologists should remain vigilant for MH throughout the perioperative period, ensure immediate access to dantrolene, and maintain appropriate postoperative monitoring in high-risk patients. This case further reinforces the importance of preparedness for atypical and delayed presentations of MH regardless of the anesthetic technique employed.
